# Comparing the Efficacy and Safety of Dexmedetomidine Versus Propofol for Sedation in Adult Patients Undergoing Cardiac Procedures: A Systematic Review

**DOI:** 10.7759/cureus.91773

**Published:** 2025-09-07

**Authors:** Shafayath Chowdhury, John Sawires, Brandon Weissman, Sera Saju, Constantino G Lambroussis

**Affiliations:** 1 Anesthesiology and Critical Care, Lake Erie College of Osteopathic Medicine, Elmira, USA; 2 Otolaryngology, Lake Erie College of Osteopathic Medicine, Elmira, USA; 3 Neurology, Lake Erie College of Osteopathic Medicine, Elmira, USA; 4 Osteopathic Medicine/Family Medicine, Lake Erie College of Osteopathic Medicine, Elmira, USA

**Keywords:** anesthesia efficacy, anesthesia safety, cardiothoracic anesthesia, hemodynamic stability, postoperative outcomes, propofol-induced anesthesia, dexmedetomidine

## Abstract

Operative cardiac procedures require meticulous sedation management to optimize patient outcomes. Two commonly used sedatives in these procedures are dexmedetomidine and propofol. Propofol is a γ-aminobutyric acid (GABA) agonist widely used for its rapid onset of action and short recovery time. Dexmedetomidine is an α₂-adrenoceptor agonist that provides sedation with minimal respiratory depression due to its opioid-sparing effect. This systematic review followed Preferred Reporting Items for Systematic Reviews and Meta-Analyses (PRISMA) guidelines to compare dexmedetomidine and propofol in adult patients undergoing cardiac procedures. A comprehensive literature search identified 648 studies that were screened down to a total of 15 that met the inclusion criteria. The included studies assessed hemodynamic stability, sedation effectiveness, postoperative recovery, incidence of adverse effects, and other relevant parameters. Findings revealed distinctive hemodynamic profiles between these two sedative agents. Dexmedetomidine administration was associated with clinically significant reductions in heart rate and mean arterial pressure, which ultimately requires vigilant monitoring for bradycardic and hypotensive episodes. In contrast, propofol demonstrated greater hemodynamic variability and was linked to higher incidences of cardiac arrhythmias as well as elevated postoperative analgesic requirements. It was also found that dexmedetomidine facilitated significantly reduced extubation times, decreased mechanical ventilator dependency, and markedly lower rates of postoperative delirium. These findings point to dexmedetomidine as a potentially advantageous option for enhanced recovery protocols in cardiac anesthesia. Propofol remains a widely used alternative due to its predictable recovery profile, but its association with increased opioid use and hemodynamic instability may limit its benefits in certain patient populations. The choice between these agents should be guided by individualized sedation strategies that balance the need for hemodynamic stability, depth of sedation, and optimization of recovery. Further research is warranted to explore long-term outcomes and refine best practices for sedation in patients undergoing cardiac procedures.

## Introduction and background

With cardiovascular disease on the rise, cardiac surgery continues to be vital in the treatment of a range of heart conditions [1]. More than one million cardiac surgeries are performed every year, making it essential to continually refine perioperative care practices [2]. Many cardiac surgery patients are older and live with chronic conditions like diabetes, kidney disease, or lung disease, all of which can complicate their recovery [3-7]. These factors make careful management in the postoperative period especially important. Sedation needs to be tailored to maintain stable vital signs and promote a smooth recovery. The choice of sedative can influence key outcomes, including how long a patient stays on a ventilator, how long they remain in the intensive care unit (ICU), and whether they experience delirium or cognitive changes postoperatively [8,9]. Beyond clinical concerns, these complications add to healthcare costs and place added pressure on ICU resources [10,11].

Postoperative sedation in cardiac surgery patients specifically presents unique challenges. Advanced age and comorbid conditions, such as diabetes, renal dysfunction, and chronic obstructive pulmonary disease (COPD), are common among this patient population [4-6]. Additionally, the use of cardiopulmonary bypass (CPB) introduces additional physiological stress, including inflammatory responses and changes in cerebral perfusion [12,13]. Such factors increase patient vulnerability to the side effects of sedation. In this population, therefore, the optimal sedative should be short-acting, easily titratable, and possess minimal potential for inducing respiratory depression, hypotension, or neurological dysfunction [14]. 

Propofol, a γ-aminobutyric acid (GABA) agonist, is widely used for ICU sedation due to its rapid onset and short half-life [15]. However, its use is restricted by drawbacks like myocardial depression, hypotension, respiratory suppression, and, in rare cases, the life-threatening propofol infusion syndrome [16,17]. These effects are further magnified in the cardiac surgery environment, where hemodynamic stability is crucial. In response to such limitations, dexmedetomidine has emerged as a promising alternative.

Dexmedetomidine is a highly selective α₂-adrenergic receptor agonist with sedative, analgesic, and sympatholytic properties [18,19]. In contrast to propofol, dexmedetomidine provides sedation that closely resembles natural sleep. This allows patients to remain arousable while still preserving respiratory drive and enabling easier neurologic evaluation [20]. It has also been associated with a lower rate of ICU delirium, shorter mechanical ventilation times, and lower opioid requirements, benefits that align with the goals of enhanced recovery after cardiac surgery [21,22].

Despite growing use of both agents, there is still no consensus on the ideal sedative for cardiac surgery patients. Individual studies have reported variable findings regarding the efficacy and safety profiles of dexmedetomidine compared to propofol. Some of these include differing outcomes in rates of delirium, ventilation duration, ICU length of stay, hemodynamic stability, and cognitive recovery. Moreover, the clinical practices often vary as per the provider’s preference, institution protocols, and patient-specific factors.

Given both the variability in reported outcomes and the absence of standardized guidelines, a systematic review was needed to evaluate the comparative efficacy and safety of dexmedetomidine and propofol in adult patients undergoing cardiac surgery. Thus, this review synthesizes current evidence on key clinical outcomes to guide evidence-based sedative selection in this high-risk population, and, through the determination of trends across existing studies, aims to support more informed and individualized sedation strategies.

## Review

Method

Design and Search Strategy

With the use of the Preferred Reporting Items for Systematic Reviews and Meta-Analyses (PRISMA) guidelines [23], a literature search was performed on PubMed and ClinicalTrials.gov from December 2024 to May 2025. Keywords used in the literature search strategy included “Propofol”, “Dexmedetomidine”, “Cardiac procedure + Propofol”, “Cardiac procedure + Dexmedetomidine”, and “Propofol versus Dexmedetomidine”. 

**Figure 1:**
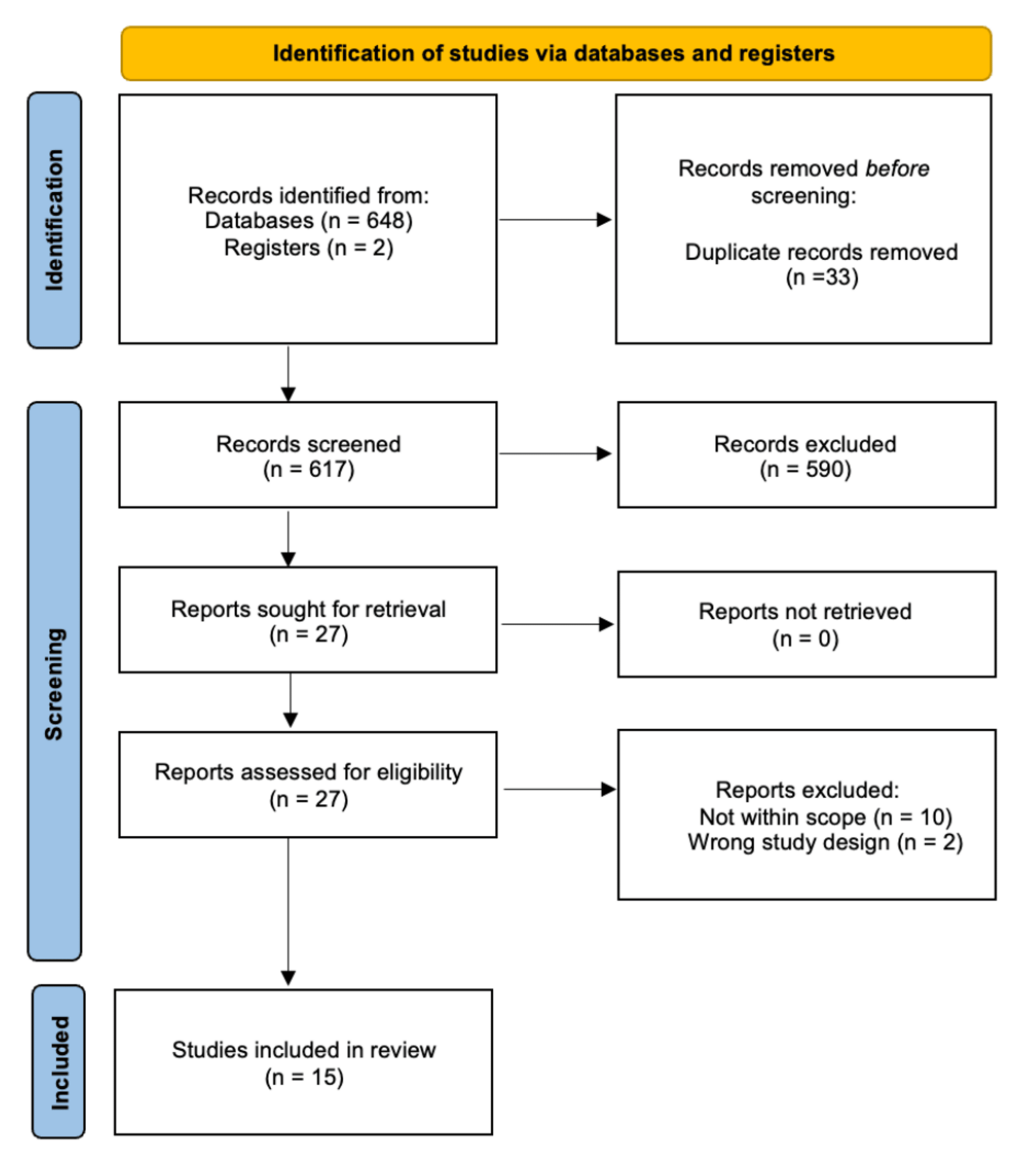
PRISMA flowchart for literature search and study selection PRISMA: Preferred Reporting Items for Systematic Reviews and Meta-Analyses

Through the literature search, 648 studies were identified initially. After the selection and screening process and application of the eligibility criteria (Table 1), a final total of 15 studies were included in this review. Figure 1 displays the PRISMA flowchart detailing the literature search and study selection process.

**Table 1:** Study selection criteria (study type) Studies that directly compared the safety and efficacy of dexmedetomidine and propofol in the context of cardiac operations in adult patients were prioritized.

Inclusion Criteria	Exclusion Criteria
Randomized Controlled Trials	Systematic Reviews
Non-randomized Controlled Trials	Meta-analyses
Prospective Trials	Commentaries/Opinions
Pilot Studies	Case Reports
Case Series	
Observational Studies	
Retrospective Studies	
Population-based Studies	
In-vivo Studies	
In-vitro Studies	
In-situ Studies	
Ex-vivo Studies	
In-silico Studies	

Results

A total of 15 studies [24-38] were included in this systematic review that compared the efficacy and safety of dexmedetomidine compared to propofol in adult patients undergoing cardiac procedures. 

In a prospective randomized pilot study, Khalil et al. (2016) compared propofol with dexmedetomidine for conscious sedation during transcatheter aortic valve implantation (TAVI) [24]. These two forms of conscious sedation were compared by their abilities to maintain hemodynamic stability, level of sedation, level of satisfaction, and the lengths of ICU or total hospital stays. The authors emphasized the importance of hemodynamic stability during TAVI due to the high-risk nature of these patients. 

Their study divided 50 patients with a mean age of 74 years, American Society of Anesthesiologists (ASA) score of 3-4, and complaints of severe aortic stenosis into a propofol group (n = 25) and a dexmedetomidine group (n = 25). The propofol group received an initial bolus of 0.5 mg/kg followed by continuous infusion at 30-50 μg/kg/min, while the dexmedetomidine group received a loading dose of 1 μg/kg followed by an infusion at 0.5 μg/kg/h. Key intraoperative measurements included heart rate, mean arterial pressure (MAP), number of phenylephrine boluses, oxygen saturation, sedation scores, and satisfaction ratings. Hemodynamic parameters were assessed before the procedure, during the cardiologic intervention, and at the end of the procedure [24].

The results of their study found a significantly increased incidence of bradycardia and hypotension and number of phenylephrine boluses in the dexmedetomidine group. There was a significant reduction in the heart rate in the dexmedetomidine group compared to the propofol group (67.28 ± 6.9 bpm vs. 78 ± 6.9 bpm, p < 0.001). MAP was also significantly lower in the dexmedetomidine group (58.12 ± 5.4 mmHg vs. 68.24 ± 11.4 mmHg, p < 0.001). Additionally, the number of phenylephrine boluses required was significantly higher in the dexmedetomidine group (36.5 ± 7.17 vs. 20.6 ± 2.07, p < 0.001), suggesting increased hemodynamic instability. However, no significant differences were found between the two groups for oxygen saturation, Ramsay Sedation Scale (RSS) scores, pain scores, patient recall of the procedure, patient satisfaction, and cardiologist satisfaction. Postoperative outcomes were also comparable between the two groups, with no significant differences in complications like renal failure, stroke, pulmonary edema, or myocardial ischemia. The length of ICU and total hospital stays were similar between the two groups as well [24].

Despite these findings, it is imperative to consider that this was a pilot study with a small sample size that needs to be performed as a larger randomized double-blinded clinical trial to better evaluate the efficacy of these drugs as conscious sedatives during TAVI. In addition, TAVI is a highly specific surgical procedure. The methods of this study need to be prospectively replicated in other major thoracic operations to compare the reliability of maintaining hemodynamic stability for both sedatives in alternative settings [24].

In a prospective randomized study, Karaman et al. (2015) compared dexmedetomidine and propofol for postoperative sedation in patients undergoing coronary artery bypass graft (CABG) surgery within a fast-track recovery room setting [25]. A fast-track recovery room setting for cardiac surgery is a streamlined process where a patient is extubated in the first six postoperative hours and often has a shorter ICU or hospital stay as a result. Their study primarily evaluated extubation times, hemodynamic stability, sedation levels, patient satisfaction, and postoperative complication rates.

In Karaman et al.'s study, a total of 64 patients scheduled for elective CABG surgery between the ages of 40 and 75, who had an ASA score of < 4, were included [25]. The patients were randomized into a group of 31 receiving dexmedetomidine infusion (0.2 - 1.0 µg/kg/h) and a group of 33 receiving propofol infusion (1.0 - 3.0 mg/kg/h). Sedation was titrated to maintain bispectral index (BIS) values between 60 and 90 and RSS scores between 3 and 4. Extubation time, sedation scores, hemodynamic parameters, and patient satisfaction were systematically recorded.

Their study found that dexmedetomidine significantly reduced extubation time compared to propofol (265.94 ± 43.1 min vs. 322.52 ± 39.2 min, p < 0.001). This suggests that dexmedetomidine is more suitable for fast-track cardiac anesthesia. RSS values were consistently higher in the dexmedetomidine group, which alluded to a deeper sedative effect. Patient satisfaction was also significantly greater with dexmedetomidine, with median scores of 9 compared to 7 in the propofol group (p < 0.001). There were no significant differences observed between the groups in terms of hemodynamic stability or postoperative adverse events like hypotension, bradycardia, or atrial fibrillation. These findings suggest that dexmedetomidine may be preferable over propofol in fast-track cardiac anesthesia due to its benefits in reducing extubation time and increasing patient satisfaction [25]. 

In a retrospective analysis, Mayr et al. (2018) compared dexmedetomidine and a propofol-opioid (PO) combination for sedation in patients undergoing transfemoral transcatheter aortic valve implantation (tf-TAVI) [26]. The study primarily assessed periprocedural gas exchange and hemodynamic support in these patients with severe aortic stenosis. A total of 297 patients were split into a group of 157 patients receiving dexmedetomidine and a group of 140 patients receiving PO sedation. The primary outcome measured was periprocedural partial pressure of carbon dioxide (PaCO₂). Secondary outcomes included incidences of hypercapnia (PaCO₂ > 45 mmHg), need for vasopressor support, conversion to general anesthesia, and the need for additional sedative “rescue therapy” in dexmedetomidine patients.

The results showed that dexmedetomidine was associated with significantly lower PaCO₂ levels compared to the PO combination (mean of 40 mmHg vs. mean of 44 mmHg, p < 0.001). In addition to lower PaCO₂ levels, the incidence of hypercapnia was also less frequent in the dexmedetomidine group compared to the PO group (25% vs. 42%, p = 0.005). Furthermore, vasopressor support was required more often in the PO group (68% vs. 25%, p < 0.001), indicating greater hemodynamic instability. There was no significant difference in conversion to general anesthesia (9% PO vs. 3% dexmedetomidine, p = 0.051). However, 16% of dexmedetomidine patients required additional sedative or opioid rescue therapy [26].

These findings suggest that dexmedetomidine may provide advantages over PO sedation for tf-TAVI by preserving respiratory function and reducing vasopressor requirements. Nevertheless, its need for supplemental sedation in some patients highlights the importance of individualized sedation strategies [26]. 

In a randomized controlled trial (RCT), Preveden et al. (2023) compared dexmedetomidine with propofol for postoperative sedation in patients undergoing elective cardiac surgery with CPB [27]. The primary objective was to assess their effects on mechanical ventilation (MV) duration, length of ICU stay, length of total hospital stay, and incidence of postoperative delirium (POD). Given the increasing focus on early recovery strategies, this study aimed to determine whether dexmedetomidine provides superior postoperative outcomes compared to propofol.

A total of 120 patients who were scheduled for elective cardiac surgery with the use of CPB were included in Preveden et al.'s study [27]. The population of patients was 18 years or older and had a left ventricular ejection fraction (LVEF) > 40%. The patients were randomized equally into two groups of 60 patients each. The dexmedetomidine group received a continuous infusion of 0.2 - 0.7 mcg/kg/h, and the propofol group was given 1 - 2 mg/kg/h. The duration of MV was significantly shorter in the dexmedetomidine group, with a reduced ventilation time of 2.2 hours on average compared to propofol (p < 0.001). A positive correlation was noted between prolonged MV duration, ICU length of stay (r = 0.368, p < 0.001), and total hospital length of stay (r = 0.204, p = 0.025). POD occurred in 25% of patients in the propofol group, but only 11.7% of patients in the dexmedetomidine group (p = 0.049). However, ICU and length of total hospital stay did not significantly differ between the groups. There were also no significant differences found in rates of atrial fibrillation or need for blood transfusions.

Despite the promising findings, this study has several limitations. The exclusion of high-risk patients, such as those with severe heart failure (LVEF < 40%) or pre-existing cognitive impairment, limits the generalizability of the results to a broader cardiac surgical population. Additionally, their study did not control for additional sedative or analgesic medications that may have influenced the outcomes [27]. 

Racman et al. (2023) conducted a randomized double-blind clinical trial comparing dexmedetomidine and propofol for procedural sedation in patients undergoing tf-TAVI [28]. Given the increasing preference for procedural sedation over general anesthesia for tf-TAVI, this study aimed to determine which sedative offers better neurocognitive and clinical outcomes. The outcomes were measured via incidence of delayed neurocognitive recovery (DNCR) and POD.

Their study included 71 patients who were 18 years or older and scheduled for tf-TAVI. The patients were split into one group of 34 receiving propofol (0.5 - 2.5 mg/kg/h) and another group of 37 receiving dexmedetomidine (loading dose of 0.5 mcg/kg followed by 0.2 - 1.0 mcg/kg/h infusion). Cognitive function was assessed using the Mini-Mental State Examination (MMSE) before and 48 hours after the procedure. The dexmedetomidine group demonstrated significantly better postoperative MMSE scores (p = 0.022) and a lower incidence of DNCR (24.3% vs. 58.8%, p = 0.005). While the incidence of POD was lower in the dexmedetomidine group (2.7% vs. 11.8%), this difference was not statistically significant (p = 0.126). Additionally, patients receiving propofol required more frequent rescue fentanyl boluses for analgesia (41.2% vs. 18.9%, p = 0.04), indicating that dexmedetomidine provided more stable sedation [28].

While the findings are compelling, Racman et al.'s study has some limitations. The sample size was relatively small, which could indicate decreased relevance of some outcomes, such as the incidence of POD. Additionally, the MMSE may not be sensitive enough to detect subtle cognitive changes postoperatively [28]. 

Djaiani et al. (2016) conducted a single-blinded RCT investigating the differences between dexmedetomidine and propofol in reducing incidences of POD after cardiac surgery [29]. The primary outcomes studied were the incidence, onset, and duration of POD. Secondary outcomes studied included hemodynamic stability, length of ICU and hospital stay, extubation times, adverse events, sedation/pain scores, and cost analysis. The 183 patients studied were randomly allocated into two groups: 91 patients in the dexmedetomidine group and 92 patients in the propofol group. The dexmedetomidine group received a 0.4 µg/kg bolus over 10-20 minutes, followed by a continuous infusion of 0.2 - 0.7 µg/kg/h limited to 24 hours. The propofol group received a continuous infusion of 25 - 50 µg/kg/min until extubation.

POD was present in 16 of 91 dexmedetomidine group patients (17.5%) and 29 of 92 propofol group patients (31.5%). In the dexmedetomidine group, 11 patients developed delirium in the ICU, and another five patients developed delirium on the surgical floor following ICU discharge. In the propofol group, 25 patients developed delirium in the ICU, and four more patients developed delirium on the surgical floor. In the dexmedetomidine group, the onset of delirium was delayed (median 2 days vs. 1 day, p = 0.027) and the duration of delirium was shorter (median 2 days vs. 3 days, p = 0.04). The incidence of major adverse outcomes, requirements for inotropic/vasoconstrictor support, and the length of stay were similar in both groups. Extubation times were slightly shorter in the dexmedetomidine group (5.4 hours vs. 5.9 hours; p = 0.0007). Sedation and pain scores were similar across groups, but opioid consumption was lower in the dexmedetomidine group (1.2 mg vs. 1.58 mg at 12 hours, p = 0.018; 4 mg vs. 5.8 mg at 24 hours, p = 0.045), and antipsychotic use was lower in the dexmedetomidine group (13% vs. 26% in the propofol group, p = 0.04). After performing a cost analysis, dexmedetomidine reduced ICU delirium-related hours and the cost of total ICU stay [29].

Their study found that dexmedetomidine reduced the incidence, delayed the onset, and shortened the duration of POD compared to propofol in elderly patients undergoing cardiac surgery. Dexmedetomidine was also associated with lower opioid/antipsychotic use and led to substantial cost savings. However, study limitations such as the single-center design, lack of blinding in sedation administration, and the restriction of dexmedetomidine infusion to 24 hours may have influenced these results. Despite these shortcomings, these findings support dexmedetomidine as a promising alternative to propofol for postoperative sedation and warrant further investigation in broader patient populations and surgical settings [29].

Liu et al. (2016) conducted a single-blinded RCT comparing the effects of dexmedetomidine versus propofol on sublingual microcirculation in adult patients undergoing elective valve surgery with CPB [30]. The primary outcome studied was the change in perfused small-vessel density (PSVD) at four hours post ICU admission (T2) compared to baseline (T1). Secondary outcomes measured included microcirculatory changes at 24 hours post ICU admission (T3), hemodynamic stability, sedation/analgesia scores, intubation time, and adverse events. 

All patients received propofol during CBP and were included in this study if they were expected to receive > 4 hours of postoperative sedation. The 68 enrolled patients were then randomly assigned to receive either dexmedetomidine (0.2 - 1.5 μg/kg/h) or propofol (5 - 50 μg/kg/min) as continuous infusions without a loading dose. Sublingual microcirculation was assessed at T1, T2, and T3 using sidestream dark-field imaging, small vessel density (SVD), proportion of perfused small vessels (PPV), and PSVD [30].

At T2, the dexmedetomidine group showed significantly greater increases in PSVD (1.3 mm/mm² vs. 0 mm/mm², p = 0.025) and trends toward higher SVD and PPV. At T3, both groups showed further improvements in microcirculation with a trend favoring dexmedetomidine in all measured parameters, but the differences were no longer statistically significant. Sedation and pain scores were similar between groups, but significantly fewer patients in the dexmedetomidine group required additional opioid analgesics in the first 24 hours (3.1% vs. 24.1%, p = 0.022). Intubation times as well as adverse events, including hypotension, bradycardia, atrial fibrillation, and delirium, were comparable between groups. There were no significant differences in hemodynamic parameters or inflammatory markers between the two groups [30].

In summary, Liu et al.'s RCT found that dexmedetomidine led to greater improvements in sublingual microcirculation compared to propofol in patients after elective valve surgery with CPB. This suggests that dexmedetomidine may enhance postoperative microvascular perfusion. Factors such as the single-center design, inclusion of only valve surgery patients, and possible spontaneous recovery of microcirculation over time limit the generalizability of the findings. Larger multicenter studies across different surgical populations may be warranted to evaluate their clinical implications. Despite these limitations, this study offers valuable insight into the potential microvascular benefits of dexmedetomidine [30].

Elgebaly and Sabry (2018) conducted an RCT comparing dexmedetomidine and propofol in mechanically ventilated patients in the ICU following open heart surgery [31]. The mechanical ventilation duration was assessed as the primary outcome. Secondary outcomes measured included length of stay in the ICU, requirements for midazolam and fentanyl as rescue medications, hemodynamic stability, and financial costs. The 50 patients were randomized to receive either dexmedetomidine (0.8 μg/kg/h) or propofol (1.5 mg/kg/h). Heart rate, mean arterial pressure, arterial blood gases, respiratory indices, and sedation scores were measured every four hours. 

Results showed that the mechanical ventilation duration and the length of stay in the ICU were similar between patients receiving dexmedetomidine and propofol. The study did find that sedation with dexmedetomidine involved higher financial costs. Dexmedetomidine patients also required lower doses of rescue medications like midazolam (5.7 ± 1.98 mg vs. 10.95 ± 3.59 mg; p < 0.001) and fentanyl (0.16 ± 0.08 mg vs. 0.29 ± 0.07 mg; p < 0.001). There were no significant differences found in arterial blood gas (ABG) values, ventilation indices, or oxygenation parameters between the two groups [31].

Their RCT found that dexmedetomidine was a safe and effective alternative to propofol for postoperative sedation in cardiovascular surgery patients, even though it may also have higher financial costs associated with its use. Dexmedetomidine offered better hemodynamic stability and lowered the requirement of rescue medications. The study may have been limited by a small sample size, lack of subgroup analysis, and use of a single-center population, but it provides valuable insight into the safety and sedative effects of dexmedetomidine in the postoperative cardiac ICU setting [31].

Maldonado et al. (2009) conducted a prospective randomized study comparing dexmedetomidine, propofol, and midazolam by incidence of POD in patients undergoing elective cardiac valve surgery with CPB [32]. Secondary outcomes included ICU and hospital length of stay, opioid requirements, and cost of care. Patients were randomized to receive postoperative sedation with dexmedetomidine (loading dose 0.4 μg/kg followed by 0.2 - 0.7 μg/kg/h infusion), propofol (25 - 50 μg/kg/min), or midazolam (0.5 - 2 mg/h). Delirium was assessed daily for three days postoperatively using diagnostic criteria and with confirmation by a neuropsychiatrist.

With the 90 patients enrolled, the incidence of delirium was lower in the dexmedetomidine group (3%) compared to the propofol (50%) and the midazolam (50%) group. Patients who developed delirium experienced significantly longer ICU stays (4.1 vs. 1.9 days, p < 0.001), prolonged hospitalizations (10.0 vs. 7.1 days, p < 0.001), and incurred higher costs of care. Fentanyl and morphine-equivalent use were significantly lower in dexmedetomidine patients compared to those receiving midazolam, but not significantly different from those receiving propofol. ICU and hospital stays were similar across groups, and no major differences in rescue antipsychotic use were observed. Cost analysis showed patients who developed delirium had nearly twice the cost compared to patients who did not develop delirium ($12,965 vs. $6,763, p = 0.004) [32].

In summary, their study found that dexmedetomidine had a lower incidence of POD compared to propofol and midazolam in valve surgery with CPB. The open-label design, lack of intention-to-treat analysis, and the high dropout rate of 24% may have imposed limitations on the study. Also, the use of a relatively young study population may underestimate the actual incidence of delirium. Regardless, this study provides strong evidence supporting dexmedetomidine’s potential in reducing delirium following cardiac surgery [32].

Chitnis et al. (2022) compared dexmedetomidine with propofol in an RCT of postoperative ICU sedation in the elderly undergoing cardiac surgery [33]. The primary outcome was quality of recovery, which was measured three days postoperatively using the Quality of Recovery questionnaire (QoR-40). The secondary outcomes included incidence of delirium, duration of delirium, time to extubation, hospital length of stay, in-hospital mortality, postoperative quality of life, and neurocognitive outcomes. Seventy patients over age 75 undergoing CABG or TAVI with CPB were randomized to receive either dexmedetomidine (0.5 - 1.5 μg/kg/h) or propofol (25 - 50 μg/kg/min) sedation. The sedation was initiated at sternal closure, then continued until normothermia and hemodynamic stability were achieved.

There were no significant differences observed in QoR-40 scores three days postoperatively between dexmedetomidine and propofol groups. The incidence of delirium was lower in the dexmedetomidine group compared to the propofol group, but the findings were statistically insignificant. There were also no significant differences in duration of delirium, time to extubation, or hospital length of stay. Neurocognitive scores three days postoperatively were only lower in the propofol group (p = 0.0005), but neurocognitive scores were similar between the two groups by six months postoperatively [33].

In conclusion, this RCT found no significant difference in early postoperative quality of recovery between dexmedetomidine and propofol as postoperative ICU sedation in patients undergoing cardiac surgery. However, dexmedetomidine may be associated with less early neurocognitive decline. Limitations of the study are its open-label design, lack of power in the secondary outcomes, subjective questionnaire use, and possible confounding due to unmeasured variables such as intraoperative fluid balance and transfusions. Despite these limitations, the study is innovative in evaluating both the quality of recovery and long-term neurocognitive function in an older population of post-cardiac surgery patients [33]. 

An RCT conducted by Herr et al. (2003) sought to compare the efficacy of sedation with dexmedetomidine versus propofol in patients after CABG in the ICU [34]. Group A consisted of 148 patients who received dexmedetomidine, while Group B consisted of 174 patients who received propofol. In their trial, the RSS was used to assess the sedation levels for each group. Other outcomes assessed were the amount of morphine used, the time alluded to when weaning off the patients, and the time to extubation. Adverse events, lab values, and vital signs were tracked to monitor the safety of each sedative.

The results showed that sedation scores were statistically insignificant between both groups (4.5 for group A vs. 4.7 for group B, p = 0.259). Patients requiring morphine varied drastically between the two groups: 72% of patients in group A did not require morphine compared to 37% in group B (p = 0.001). The dexmedetomidine group patients were extubated 223 minutes earlier than the propofol group. There was also a greater increase in blood pressure in the propofol group compared to the dexmedetomidine group (9 mmHg above baseline, p = 0.018) [34].

Adverse events were present in both groups observed in this study. Dexmedetomidine was more likely to cause hypertension (19 incidents) compared to propofol (6 incidents). There was also a similar trend with hypotension being more associated with dexmedetomidine (24% of patients) compared to propofol (16% of patients). Ventricular tachycardia occurred more frequently in the propofol group (5% of patients), but no patients in the dexmedetomidine group experienced ventricular tachycardia (p = 0.007). The dexmedetomidine group required fewer postoperative medications, including β-blockers, antiemetics, nonsteroidal anti-inflammatory drugs, epinephrine, and high-dose diuretics. The authors concluded that dexmedetomidine provided safer and more effective sedation after CABG surgery compared to propofol [34].

In an RCT by Mogahd et al. (2017), 70 patients aged 40 - 60 years old with an LVEF > 40% underwent CABG [35]. These patients were randomly assigned to a ketamine-dexmedetomidine group or a ketamine-propofol group. RSS was used to access the sedation level in each group. Postoperative pain was assessed using the adult nonverbal pain score. Fentanyl was given for postoperative analgesia based on the patient's adult nonverbal pain score. The sedation combinations in each group were compared based on fentanyl consumption, time to weaning from mechanical ventilation, time to extubation, and length of ICU stay.

The ketamine-dexmedetomidine and ketamine-propofol groups had insignificant variations in MAP, heart rate, and length of ICU stay. However, notable differences emerged in ventilation and analgesic outcomes. Weaning and extubation times were significantly shorter in the ketamine-dexmedetomidine group (p < 0.001 and p < 0.0001, respectively). Furthermore, this group required considerably less fentanyl in the first 24 hours postoperatively (41.94 μg vs. 152.8 μg, p < 0.0001). This points towards superior analgesic properties of dexmedetomidine when used in combination with ketamine [35].

Overall, these findings support the use of the ketamine-dexmedetomidine combination as the preferred sedation strategy in post-CABG patients due to significantly shorter mechanical ventilation times and less fentanyl dose requirements. Nonetheless, the study may be limited by its relatively small sample size, single-center design, and narrow patient demographic (ages 40-60 with preserved LVEF). This may limit generalizability to broader cardiac surgery populations such as those with reduced cardiac function or older age. Additionally, the lack of long-term outcome data and reliance on subjective pain assessment tools could introduce bias. Despite these limitations, the trial highlights the potential of dexmedetomidine-based regimens to optimize postoperative recovery by minimizing opioid use and facilitating earlier extubation [35].

Sheikh et al. (2018) carried out a double-blinded clinical RCT that aimed to compare dexmedetomidine and propofol during cardiac surgery on different hemodynamic variables and postoperative outcomes over a duration of two years [36]. Sixty patients were randomly placed into two groups of 30, either receiving dexmedetomidine or propofol after induction of anesthesia. Patients in the propofol group received a continuous infusion of propofol while patients in the dexmedetomidine group received a bolus followed by continuous infusion. Vasopressor and inotropes were used during surgery to maintain heart rate and MAP. The length of ICU stay and duration of postoperative ventilation were measured, and the risk of adverse events such as delirium was compared between each group. 

The results showed that in both groups, heart rate fluctuated in the prebypass period until the first 30 minutes postbypass. However, the mean heart rate was significantly higher in the propofol group compared to the dexmedetomidine group (p < 0.001). MAP was also found to be significantly higher in the propofol group during the prebypass period (p < 0.001). Both groups experienced a similar fall in MAP during the start of the procedure and a subsequent rise after termination of the procedure. There were no differences in the requirements of inotropes and vasopressors in either group. Patients in the dexmedetomidine group required a significantly shorter duration of postoperative ventilation compared to the propofol group (5.916 ± 4.30 hr vs. 8.566 ± 4.24 hr, p = 0.019). Similarly, the duration of ICU stay was also shorter in the dexmedetomidine group (92.80 ± 40.71 hr vs. 133.46 ± 48.73 h, p < 0.019). The incidence of delirium was significantly less in the dexmedetomidine group (p = 0.022). Overall, dexmedetomidine was superior to propofol in maintaining hemodynamic stability. It was also effective for decreasing the risk of adverse events such as delirium and allowing for shorter ICU stays [36]. 

Shi et al. (2019) investigated the effects of dexmedetomidine on POD in a randomized, double-blinded, and placebo-controlled clinical trial [37]. The study included 164 patients > 60 years old who underwent cardiac surgery receiving general anesthesia via maintenance with dexmedetomidine or propofol. The Confusion Assessment Method (CAM) for ICUs was used to assess the incidence of POD in each group. These assessments were done twice a day, beginning around 24 hours after surgery, until the fifth day after surgery. Comorbidities and preoperative medications were similar between each group. Other postoperative outcomes in patients experiencing delirium were also measured, such as delirium onset and day, extubation time, ICU length of stay, and total hospital length of stay.

Incidence of POD was statistically insignificant when comparing the two groups, where 33 of 84 patients in the dexmedetomidine group and 21 of 80 patients in the propofol group experienced POD. Of the patients experiencing POD, the onset of delirium was decreased in the dexmedetomidine group compared to the propofol group. The duration of delirium was also reduced in the dexmedetomidine group (2 days vs. 3 days). The dexmedetomidine group also required less morphine than the propofol group. Factors such as ICU stay time, hospital stay time, inotropic/vasoconstrictor support, and blood transfusions were similar between both groups [37]. 

Although the incidence of POD was similar between both groups, Shi et al. concluded that perioperative dexmedetomidine administration is more beneficial in elderly patients undergoing cardiac surgery due to the delayed onset and shorter duration of delirium. However, the study has some limitations that should be considered. The diagnosis of POD relied on intermittent CAM assessments, which may miss transient episodes of delirium between evaluations. Additionally, the trial did not explore long-term cognitive outcomes or functional recovery, which are critical in evaluating the full impact of POD interventions in elderly patients. The generalizability of the findings may also be limited since the study population included only patients over 60 years of age and was conducted in a single healthcare setting. Despite these limitations, the findings reinforce the role of dexmedetomidine in postoperative care as a strategy not only for sedation and opioid reduction, but also for modulating the neuroinflammatory cascade that contributes to delirium [37]. 

Metry et al. (2019) conducted a prospective, randomized, double-blind study to compare the effects of propofol versus dexmedetomidine on cerebral regional oxygen saturation (rSO₂) in adult patients undergoing CPB procedures [38]. The study included 50 patients undergoing elective cardiac surgery who were evenly randomized into two groups: one receiving dexmedetomidine infusion (1 μg/kg loading dose followed by 0.2 - 0.7 μg/kg/h) and the other receiving propofol infusion (2 - 3 mg/kg/h). Both groups were maintained under general anesthesia using a standardized regimen including fentanyl, isoflurane, and muscle relaxants. Cerebral oxygenation was continuously monitored using near-infrared spectroscopy at eight time points: before induction (T0), 10 minutes post-induction (T1), five minutes after initiation of CPB (T2), at target hypothermia (T3), during CPB at 30 and 60 minutes (T4 and T5), after rewarming (T6), and five minutes after weaning from CPB (T7). Postoperative cognitive function was assessed using the MMSE at baseline and five days postoperatively.

The study found no statistically significant differences in cerebral regional oxygen saturation between the dexmedetomidine and propofol groups at any measured time point during the perioperative period. Both agents maintained stable cerebral oxygenation profiles throughout CPB. Additionally, MMSE scores postoperatively did not differ significantly between the two groups, suggesting no meaningful difference in early postoperative cognitive function. The authors concluded that fluctuations in rSO₂ during CPB were more closely associated with physiological variables such as hematocrit, pH, PaCO₂, and core temperature instead of the choice of sedative agent [38].

Their RCT demonstrated that both dexmedetomidine and propofol preserve cerebral oxygenation during cardiac surgery with CPB and have comparable effects on early cognitive function. However, the study is limited by its small sample size, short duration of postoperative cognitive follow-up, and the use of MMSE. The single-center design and lack of blinding for intraoperative management may also introduce potential bias. Despite these limitations, the study adds to the growing body of literature assessing neuroprotective strategies in cardiac anesthesia and underscores the importance of optimizing physiological parameters over specific anesthetic choice to maintain cerebral perfusion during CPB [38].

Table 2 presents the key findings from the included studies that directly compared dexmedetomidine and propofol in terms of their efficacy and safety as sedatives used for adult patients undergoing cardiac procedures. 

**Table 2:** Summary of findings from the included studies comparing propofol with dexmedetomidine TAVI: transcatheter aortic valve implantation; CABG: coronary artery bypass graft; tf-TAVI: transfemoral transcatheter aortic valve implantation; CPB: cardiopulmonary bypass; IV: intravenous; ICU: intensive care unit; MAP: mean arterial pressure; HR: heart rate

Authors (year)	Study Design	Sample Size	Type of procedure	Route of administration	Key Findings
Khalil et al., 2016 [24]	Prospective randomized pilot study	50	TAVI	IV infusion	Dexmedetomidine significantly increased bradycardia, hypotension, and phenylephrine use compared to propofol during TAVI. Both groups had comparable sedation, patient satisfaction, and postoperative complication rates, with similar lengths of ICU/hospital stays.
Karaman et al., 2015 [25]	Prospective randomized study	64	CABG	IV infusion	Dexmedetomidine significantly reduced extubation time and enhanced patient satisfaction compared to propofol in patients undergoing fast-track CABG surgery. Hemodynamic stability and rates of adverse events were similar between the two sedatives.
Mayr et al., 2018 [26]	Retrospective analysis	297	tf-TAVI	IV infusion	Dexmedetomidine demonstrated superior respiratory stability (lower PaCO₂ and fewer episodes of hypercapnia) and reduced vasopressor requirements compared to propofol-opioid sedation in patients undergoing tf-TAVI. However, additional sedative rescue therapy was more frequently required with dexmedetomidine.
Preveden et al., 2023 [27]	Randomized controlled trial	120	Elective cardiac surgery with CPB	IV infusion	Dexmedetomidine significantly shortened mechanical ventilation duration compared to propofol after elective cardiac surgery. Although postoperative delirium occurred less frequently with dexmedetomidine, ICU and hospital stay durations did not differ significantly between groups.
Racman et al., 2023 [28]	Randomized double-blind clinical trial	71	tf-TAVI	IV infusion	Dexmedetomidine significantly improved postoperative cognitive outcomes and reduced the incidence of delayed neurocognitive recovery compared to propofol sedation during TAVR. Dexmedetomidine also decreased the requirement for additional analgesic rescue therapy.
Djaiani et al., 2016 [29]	Randomized controlled trial	183	Cardiac surgery	IV infusion	Dexmedetomidine significantly reduced the incidence, delayed onset, and shortened duration of postoperative delirium compared to propofol. The absolute risk reduction for POD was 14%, with a number needed to treat of 7.1
Liu et al., 2016 [30]	Randomized controlled trial	68	Elective valve surgery with CPB	IV infusion	Dexmedetomidine significantly improved sublingual microcirculation compared to propofol postoperatively with a higher perfused small-vessel density.
Elgebaly and Sabry, 2018 [31]	Randomized controlled trial	50	CABG	IV infusion	Dexmedetomidine was a safe and effective alternative to propofol, but was associated with higher costs. Dexmedetomidine also offered better hemodynamic stability and lowered the requirement of rescue medications
Maldonado et al., 2009 [32]	Open-label randomized clinical trial	90	Elective valve surgery with CPB	IV infusion	Dexmedetomidine significantly reduced the incidence of postoperative delirium compared to propofol and midazolam. Analysis showed patients who developed delirium had nearly twice the cost and longer ICU stays compared to patients who did not develop delirium
Chitnis et al., 2022 [33]	Randomized controlled trial	70	CABG or TAVI	IV infusion	Dexmedetomidine and propofol showed no difference in early postoperative quality of recovery, but dexmedetomidine was associated with less early neurocognitive decline.
Herr et al., 2003 [34]	Randomized controlled trial	322	CABG	IV infusion	Dexmedetomidine provided safer and more effective sedation after CABG surgery compared to propofol. The dexmedetomidine group had a shorter time to extubation and was less likely to require morphine while decreasing likelihood of adverse events.
Mogahd et al., 2017 [35]	Randomized controlled trial	70	CABG	IV infusion	Sedation combination with ketamine-dexmedetomidine led to significantly shorter mechanical ventilation times and less fentanyl dose requirements in CABG surgery compared to sedation with a combination of ketamine-propofol. However, there were insignificant differences between MAP, HR and length of ICU stay between both groups.
Sheikh et al., 2018 [36]	Randomized controlled, double-blind, clinical trial	60	CPB	IV infusion	Perioperative infusion with dexmedetomidine is preferred compared to propofol due to significantly shorter duration of postoperative ventilation and ICU stay. MAP and HR were higher with propofol infusion. Delirium occurred significantly less in the dexmedetomidine group. There were no differences in the requirements of inotropes or vasopressors in either group.
Shi et al., 2019 [37]	Randomized, double-blind, and placebo-controlled clinical trial	164	Cardiac surgery	IV infusion	Perioperative dexmedetomidine administration is more beneficial in elderly patients undergoing cardiac surgery compared to propofol due to delayed onset and shorter duration of delirium. However, incidence of postoperative delirium is statistically insignificant when receiving general anesthesia maintenance of dexmedetomidine and propofol.
Metry et al., 2019 [38]	Prospective, randomized, double-blind study	50	CPB	IV infusion	There were no differences between propofol and dexmedetomidine on cerebral regional oxygen saturation before, during and after CPB procedures. This suggested that there is no difference in neurologic outcome with either drug.

Discussion

Effectiveness of sedation is a crucial component of anesthetic management in cardiac surgery. Literature has continuously found that outcomes related to depth of sedation, hemodynamics, postoperative adverse events, and delirium in cardiac surgery can be greatly influenced by the effectiveness of sedation. Propofol and dexmedetomidine are considered first-line agents for sedation from updated guidelines by the Critical Care Medicine and the American Society of Health-System Pharmacists in 2012 [39].

Dexmedetomidine: A Selective α₂-Adrenoceptor Agonist

Dexmedetomidine is a highly selective α₂-adrenoceptor agonist introduced into clinical practice for its sedative and analgesic properties. The drug was chosen for use mainly in intensive care settings and for procedural sedation. Its high selectivity for α₂-receptors (α₂:α₁ ratio of 1620:1) distinguishes it from other agents like clonidine (α₂:α₁ ratio of 220:1) [40].

The sedative effects of dexmedetomidine are mediated through activation of α₂-adrenoceptors in the locus coeruleus of the brain. This activation leads to inhibition of norepinephrine release and promotion of a sedative state that closely resembles natural sleep. This mechanism allows for sedation without significant respiratory depression, which gives dexmedetomidine a notable advantage over other sedatives ​[41]. Activation of presynaptic α₂-adrenoceptors inhibits the release of norepinephrine, thereby attenuating pain signal transmission. Additionally, postsynaptic activation within the central nervous system reduces sympathetic activity and results in decreased blood pressure and heart rate [42].

Pharmacokinetically, dexmedetomidine exhibits a rapid distribution phase and is extensively metabolized in the liver, with metabolites excreted in the urine. Its context-sensitive half-life allows for relatively predictable recovery times, which makes it suitable for both short-term and prolonged sedation [43]. Common adverse effects include bradycardia and hypotension due to its sympatholytic properties. However, its minimal impact on respiratory function and its ability to provide cooperative sedation make it a valuable agent in various clinical scenarios [44].

Propofol: A Short-Acting Intravenous Anesthetic

Propofol is a widely utilized intravenous hypnotic agent employed for the induction and maintenance of general anesthesia as well as for sedation in various medical procedures. Its rapid onset and short duration of action make it particularly suitable for procedures requiring swift recovery times [45]. The primary mechanism of action of propofol involves potentiation of the inhibitory neurotransmitter GABA at the γ-aminobutyric acid type A (GABAA) receptor. By enhancing GABAA receptor activity, propofol increases chloride ion conductance, leading to hyperpolarization of neuronal membranes and subsequent inhibition of neuronal firing [46].

In terms of pharmacokinetics, propofol is characterized by rapid distribution and clearance. It is highly lipophilic and thus results in a swift onset of sedation within 30-60 seconds. The drug undergoes extensive hepatic metabolism via conjugation, and its metabolites are excreted renally. The context-sensitive half-life of propofol is relatively short and contributes to its favorable recovery profile [47].

Hemodynamic effects of propofol include dose-dependent hypotension, mostly due to systemic vasodilation and negative inotropic effects. Respiratory depression is another notable side effect that necessitates careful monitoring during administration. Despite these effects, propofol is favored for its antiemetic properties and minimal residual sedation. These features prove to be advantageous in both surgical and diagnostic settings [48]. 

This study aimed to compare the effectiveness of dexmedetomidine with that of propofol in adult patients who underwent cardiac procedures such as CABG, TAVI, and other procedures requiring CPB. This systematic review focused on hemodynamic stability, the effectiveness of sedation, ventilation parameters, and the incidence of POD. With the rise of fast-track cardiac care, understanding effective anesthesia protocols is crucial for early extubation and minimizing the use of opioids during cardiac procedures [49]. 

Hemodynamic Stability

A critical aspect of cardiac surgery is the ability to maintain hemodynamic stability to avoid intraoperative complications, as well as to maximize outcomes. Numerous studies have observed a significant difference in hemodynamic stability between dexmedetomidine and propofol. Dexmedetomidine led to significant decreases in heart rate and MAP compared to propofol. This is an expected finding due to the reduction of sympathetic neurotransmitter release by dexmedetomidine [24,36]. Although the mechanism of action of dexmedetomidine may be beneficial in decreasing myocardial oxygen demand, it also increases instances of bradycardia, hypotension, and the need for vasopressors like phenylephrine [24]. In contrast, propofol leads to vasodilation and myocardial depression, which can also lead to hypotension. Propofol was also found to increase the likelihood of ventricular tachycardia due to its effects on the baroreceptor reflex [34]. The effects of each propofol and dexmedetomidine on hemodynamics differ greatly, and the choice of drug may differ based on individual clinical status.

Effectiveness in Sedation 

Dexmedetomidine and propofol are effective sedatives that are commonly utilized in clinical practice. Several studies have analyzed differences in their sedative properties. When using the RSS, some studies found no statistical significance when comparing patients who were administered each drug, suggesting that both propofol and dexmedetomidine provide adequate sedation in cardiac procedures [24,34]. However, Sheikh et al. found that RSS scores were higher in patients who were administered dexmedetomidine compared to propofol, suggesting that dexmedetomidine may cause deeper sedation. In this study, patient satisfaction was significantly greater with dexmedetomidine [36]. Additionally, the need for a rescue opiate or an additional sedative was more likely to occur in patients administered propofol for sedation compared to dexmedetomidine. As a result, the use of dexmedetomidine may be beneficial in many patient populations by possibly avoiding opioid-related side effects via a greater analgesic sparing benefit [28,31]. 

Postoperative Outcomes

There is substantial research regarding postoperative outcomes when dexmedetomidine and propofol are administered during cardiac surgery. For instance, several studies have found that dexmedetomidine leads to significantly reduced weaning and extubation time compared to propofol. This suggests dexmedetomidine to be more suitable for fast-track cardiac anesthesia by allowing patients to be extubated in the first six postoperative hours and leading to increased patient satisfaction while also decreasing length of hospital stay [25,33-35]. Additionally, patients administered dexmedetomidine had significantly shorter durations of mechanical ventilation compared to propofol [27,35]. These findings suggest that, following cardiac procedures, dexmedetomidine has the potential to lead to faster recovery times while avoiding respiratory complications. 

Postoperative Delirium, Cerebral Oxygenation, and Microcirculation

Postoperative delirium leads to confusion and disorientation in many elderly patients. Investigating the incidence of delirium between different sedatives is important to improve patient outcomes. When investigating whether dexmedetomidine or propofol would reduce postoperative delirium, researchers found that perioperative dexmedetomidine administration led to a lower incidence, delayed onset, and shorter duration of delirium compared to propofol following cardiac procedures [29,32,33,37]. Additionally, Metry et al. compared outcomes on cerebral regional oxygen saturation between propofol and dexmedetomidine and found no differences at any time period after infusion [38]. Furthermore, Liu et al. found that dexmedetomidine led to greater improvements in sublingual microcirculation compared to propofol, suggesting greater postoperative microvascular perfusion [30]. These findings suggest that dexmedetomidine could be preferred for use in populations at higher risk for postoperative delirium, due to its strengths in neurocognitive protection and improved microcirculation.

Clinical Decision-Making and Cost-Effectiveness

The anesthetic team should consider multiple factors when choosing between propofol and dexmedetomidine. This choice should be specifically tailored to the patient's hemodynamic profile and comorbidities. Dexmedetomidine should be the preferred choice in patients who have a history of opioid dependence, pulmonary disease, or are at high risk of delirium. Propofol should be considered when rapid titration and offset of sedation are implicated, as well as in patients with chronic bradycardia. While dexmedetomidine may be the most effective sedative in many patient populations, its higher cost compared to propofol is a limiting factor in many clinical settings. In contrast, Elgebaly and Sabry found that use of dexmedetomidine leads to shorter ICU stays and ventilation times as well as a greater analgesic-sparing benefit, which can decrease the total cost in centers using a fast-track cardiac care model [31]. Future research can assess long-term cost effectiveness, including postoperative visits and long-term complications.

*Limitations and Future Directions* 

This review is subject to many limitations. Many of the studies included small sample sizes that limited statistical power. Common sedation scoring, such as RSS, is limited in terms of assessing the quality of anesthesia. Long-term adverse events and outpatient follow-ups were not assessed in the majority of the studies, leading to inadequate assessment of many relevant long-term adverse events as well as overall patient satisfaction. Future research should include RCTs in multiple clinical settings with significant statistical power. This research should emphasize long-term complications and how they relate to the use of opioids and the overall quality of life of these patients postoperatively. Effects on neurologic recovery, biomarkers of inflammation, and endothelial dysfunction should also be studied. An emphasis on standardizing research protocols to assess different aspects of sedation intraoperatively is important for accuracy and consistency between different research models.

## Conclusions

This systematic review synthesizes current evidence comparing the efficacy and safety of dexmedetomidine and propofol for sedation in adult patients undergoing cardiac procedures. Dexmedetomidine was associated with improved hemodynamic stability, shorter extubation/mechanical ventilation times, and reduced rates of POD when compared to propofol. Although dexmedetomidine requires careful monitoring due to potential bradycardia and hypotension, its opioid-sparing effects and favorable neurocognitive outcomes suggest it to be particularly advantageous in fast-track cardiac anesthesia. Propofol remains a viable option due to its predictable pharmacokinetic profile, but increased opioid requirements and greater hemodynamic variability may limit its applicability in certain patient populations. Future research focusing on larger scale studies and diverse patient populations will be crucial to refining sedation protocols and optimizing patient outcomes. Continued interdisciplinary collaboration and innovative approaches will help refine strategies in the use of propofol or dexmedetomidine for cardiac procedures requiring anesthetic sedation. 
