# Expression of Concern: Comparing the Efficacy and Safety of Dexmedetomidine Versus Propofol for Sedation in Adult Patients Undergoing Cardiac Procedures: A Systematic Review

**DOI:** 10.7759/cureus.x59

**Published:** 2025-11-19

**Authors:** Shafayath Chowdhury, John Sawires, Brandon Weissman, Sera Saju, Constantino G Lambroussis

**Affiliations:** 1 Anesthesiology and Critical Care, Lake Erie College of Osteopathic Medicine, Elmira, USA; 2 Otolaryngology, Lake Erie College of Osteopathic Medicine, Elmira, USA; 3 Neurology, Lake Erie College of Osteopathic Medicine, Elmira, USA; 4 Osteopathic Medicine/Family Medicine, Lake Erie College of Osteopathic Medicine, Elmira, USA

The Editors-in-Chief are issuing this Expression of Concern to inform readers that this systematic review incorporates data from reference #37 (Shi 2019), which was retracted in 2022. The inclusion of retracted material may compromise the reliability of the evidence synthesis and may affect the validity of the review’s conclusions. This notice is provided to ensure transparency and to assist readers in interpreting the findings appropriately.

